# Molecular Mechanism of Dietary Restriction in Neuroprevention and Neurogenesis: Involvement of Neurotrophic Factors

**DOI:** 10.5487/TR.2008.24.4.245

**Published:** 2008-12-01

**Authors:** Hee Ra Park, Mikyung Park, Hyung Sik Kim, Jaewon Lee

**Affiliations:** Department of Pharmacy, College of Pharmacy and Research Institute for Drug Development, Longevity Life Science and Technology Institutes, Pusan National University, Geumjeong-gu, Busan, 609-735 Korea

**Keywords:** Aging, Brain, Caloric restriction, Neurodegenerative diseases, Neurogenesis

## Abstract

Dietary restriction (DR) is the most efficacious intervention for retarding the deleterious effects of aging. DR increases longevity, decreases the occurrence and severity of age-related diseases, and retards the physiological decline associated with aging. The beneficial effects of DR have been mostly studied in non-neuronal tissues. However, several studies have showed that DR attenuate neuronal loss after several different insults including exposure to kainate, ischemia, and MPTR Moreover, administration of the non-metabolizable glucose analog 2-deoxy-D-glucose (2DG) could mimic the neuroprotective effect of DR in rodent, presumably by limiting glucose availability at the cellular level. Based on the studies of chemically induced D.R., it has been proposed that the mechanism whereby DR and 2DG protect neurons is largely mediated by stress response proteins such as HSP70 and GRP78 which are increased in neurons of rats and mice fed a DR regimen. In addition, D.R., as mild metabolic stress, could lead to the increased activity in neuronal circuits and thus induce expression of neurotrophic factors. Interestingly, such increased neuronal activities also enhance neurogenesis in the brains of adult rodents. In this review, we focus on what is known regarding molecular mechanisms of the protective role of DR in neurodegenerative diseases and aging process. Also, we propose that DR is a mild cellular stress that stimulates production of neurotrophic factors, which are major regulators of neuronal survival, as well as neurogenesis in adult brain.

## INTRODUCTION

The average life span of humans has increased markedly since prehistoric times as a result of improved health care, whereas the maximal life span has remained largely unchanged. Therefore, one may presume that the average life span can be extended by improving environmental conditions, while the maximal life span is thought to be increased by actually decreasing the rate of aging. McCay *et al.* first reported that limiting food intake increased the longevity in rats (McCay *et al.*, 1989). Since that seminal report, the anti-aging actions of dietary restriction (DR) have been confirmed and reconfirmed repeatedly. Today, DR is widely recognized as the most effective and repeatable means of altering and slowing the aging processes. If animals are allowed 25 to 40% less food than their *ad libitum* (AL) intake, their average life span and maximal life span are extended by about the same degree of reduced diet intake, and this is associated with a slower rate of mortality and delayed onset of age-related pathologies. In this typical type of study, animals in a DR regimen are fed once per day with a 40% reduced calorie intake. However, similar increases in both the average and maximal life span are obtained in animals fed on an every-other-day schedule, in which animals will consume approximately 20% fewer calories over time than do animals fed every day (Duffy *et al.*, 1997; Weindruch *et al.*, 1988). The hypothesis that DR can reduce risk for age-related neurodegenerative disorders has been lately raised and data show that DR protect neurons in several different age-related neurodegenerative diseases models including Alzheimer's diseases, Huntington's diseases, Parkinson's diseases, and stroke

(Prolla and Mattson, 2001). Due to the lack of proper cellular model system for DR study, the cellular mechanisms for the life-extending actions of DR have not been established clearly. In the previous trial of the chemically-induced DR, administration of the non-metabolizable glucose analog 2-deoxy-D-glucose (2DG) to rats and mice mimics the neuroprotective effect of DR, presumably by limiting glucose availability at the cellular level (Duan and Mattson, 1999; Lee *et al.*, 1999b; Yu and Mattson, 1999). In this review, we focus on what is known regarding a molecular and mechanistic understanding of the protective role of DR in neurodegenerative diseases and aging process. Additionally, we propose that DR is a mild cellular stress that induces production of neurotrophic factors, which are major regulators of neuronal survival, as well as neurogenesis in adult brain.

***Anti-aging and neuroprotective effects of DR.*** DR has been shown to reduce the development of age-related cancers and alteration in the immune system, and to decrease levels of cellular oxidative stress in several organ systems (Sohal and Weindruch, 1996). The majority of such studies have focused on non-neural tissues, and few studies have examined the effects of DR on aging brain. However, recent research has documented “anti-aging” and neuroprotective effects of DR on the brain. Several studies have suggested that DR can retard the development of spontaneous age-related changes (Major *et al.*, 1997; Morgan *et al.*, 1997). For instance, age-related increases in levels of protein oxidation (Ayala *et al.*, 2007; Dubey *et al.*, 1996) and glial fibrillary acidic protein (Major *et al.*, 1997; Sharma and Kaur, 2008) in the brain are suppressed in rats maintained on DR. Both rats and mice maintained on DR perform better on learning and memory tasks than do animals fed AL (Adams *et al.*, 2008; Roberge *et al.*, 2008). In addition to slowing the aging process, DR reduces neuronal damage and improve behavioral outcome in experimental animal models relevant to the pathogenesis of Alzheimer’s disease (Mattson, 2000), Parkinson’s disease (Duan and Mattson, 1999), Huntington’s disease (Duan *et al.*, 2003), and stroke (Mattson *et al.*, 2000). Furthermore, epidemiological data from longitudinal studies suggest that individuals with a low calorie intake are at reduced risk for Parkinson’s disease (Mattson, 2003) and Alzheimer’s disease (Patel *et al.*, 2005; Wang *et al.*, 2005). Moreover, there appears to be an inverse relationship between per capita food consumption and risk of the age-related neurodegenerative disorders among the different populations throughout the world.

***Molecular Mechanisms involving stress response proteins and neurotrophic factors.*** The free radical theory of aging postulates that oxidative stress-induced damage to cellular components is a causal factor in aging and shortening of life span (Yu, 1996). Oxidative stress, which can arise from an imbalance in the production of reactive oxygen species (ROS) and a decline of antioxidant defenses, is currently thought to respond to DR (Lopez-Torres and Barja, 2008; Sohal and Weindruch, 1996; Weindruch and Sohal, 1997). It has been shown that DR provides several protective mechansisms against oxidative damages and, at the same time, increases antioxidant defense systems including major defensive enzymes and major ROS scavengers during the aging process (Frame *et al.*, 1998; Yu and Chung, 2001a, b). These results suggest that DR exerts diverse benefits through the ability to regulate redox balance. It has also been evidenced that the redox-sensitive transcription factors involved in ROS generation and inflammation are up-regulated during aging, and interestingly down-regulated by DR (Chung *et al.*, 2001; Kim *et al.*, 2002; Lee *et al.*, 1999a). Therefore, this theory is widely accepted in justifying DR works in retarding aging process because it can explain the complexities of aging by its complicated and broad actions in aerobic living life.

However, it is clear that DR, as a mild metabolic stress, modifies neuroendocrine functions and fuel metabolism, via cellular and molecular changes within physiological limits. Aforementioned research papers have shown that the levels of stress response proteins (SRPs) such as HSP70 and GRP78 are induced in animals maintained on a DR regimen, and suggest that SRPs play an important role in protecting neurons against insults (Lee *et al.*, 1999b; Mattson, 2008; Prolla and Mattson, 2001; Yu and Mattson, 1999). Although the exact roles of SRPs in the aging process are not yet clear, SRPs are known to help the cell disassemble and dispose of damaged proteins, and to facilitate the making and transport of new proteins.

Neurotrophic factors (NTFs) are one class of signaling molecules that appears to play a prominent role in promoting the survival and growth of neurons. NTFs are defined as a specific set of proteins produced and released by cells within the nervous system that act on specific cell surface receptors linked to signal transduction pathway that promotes neuronal survival and outgrowth, and protect neurons against insults. Many different NTFs are greatly increased in response to brain injury, and they appear to represent prototypical anti-oxidation intercellular messengers in the brain (Mattson *et al.*, 1995). Therefore, they are believed to

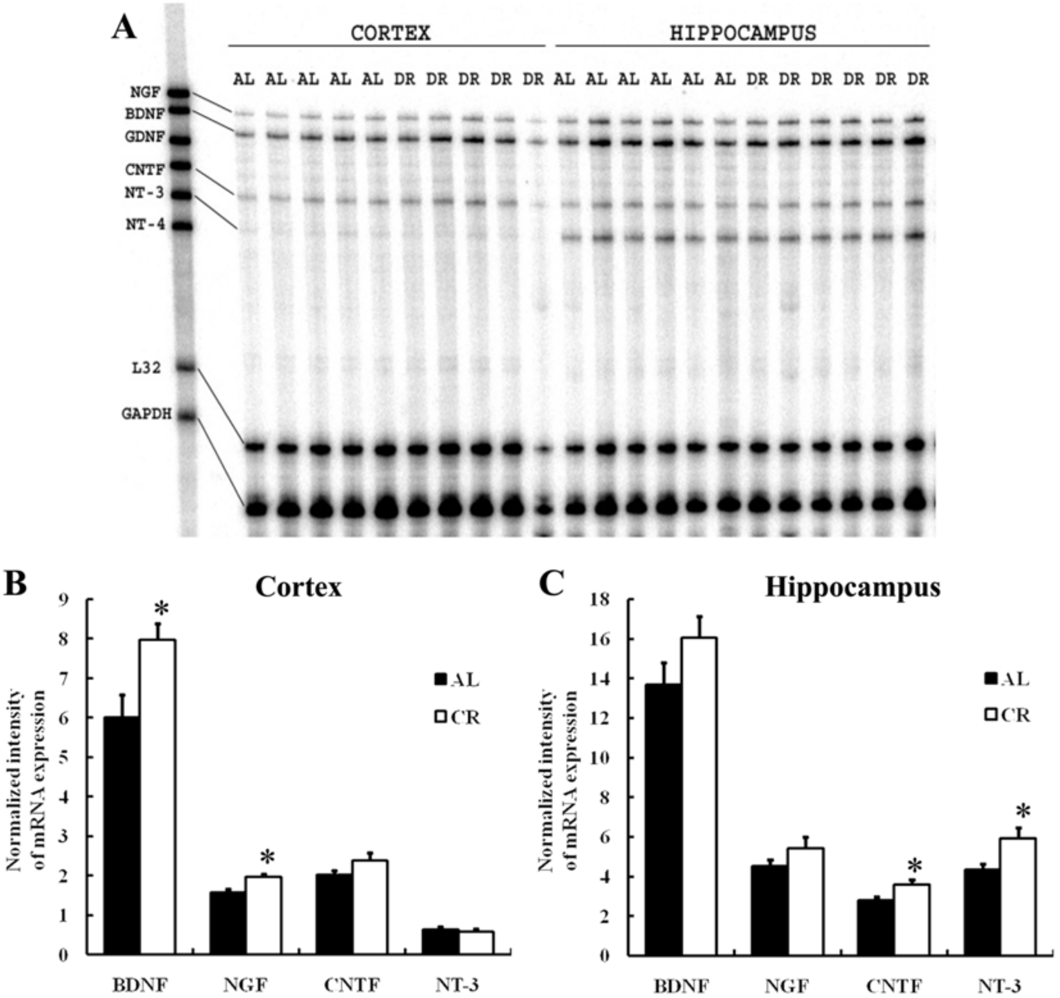

**Fig. 1.** RNase protection assay for expression of neurotrophic factors in mRNAs isolated from cortex and hippocampus of rats fed on AL or DR regimen for 3 months. (A) Total RNA in hippocampal and cortical tissue homogenates from rats in groups AL and DR were hybridized to multi-riboprobes of NTFs (rNT-1 from RiboQuant$^{TM}$). Protected RNA fragments were analyzed on denaturing acrylamide gels, which were exposed to PhosphoImager screen and scanned. (B~C) Quantitative assessment of NTF mRNA levels in cortex (B) and hippocampus (C). Values for mRNA levels obtained from RNase protection assays are expressed as the normalized value after correction for corresponding levels of GAPDH mRNAs. Values are the mean and standard error ($n = 5$ or 6 rats/group). $*p < 0.05$ compared to corresponding value for AL group (ANOVA with Fisher's protected least significant difference procedure).

be an intrinsic defense mechanism in response to oxidative stress that is a common correlate of cell damage and organ dysfunction in normal aging and age-related degenerative disorders. The current data showed that DR up-regulated several NTFs in the hippocampus and cortical neurons transcriptionally (Fig. 1 & Table 1) as well as translationally (Duan *et al.*, 2001; Lee *et al.*, 2000). The upregulation of NTF were generally observed in the brain injury condition. Interestingly, NT-3 has been known to be down-regulated in the hippocampus following brain injury such as seizures (Lowenstein and Arsenault, 1996; Rocamora *et al.*, 1992). However, DR-mediated upregulation of NTFs were not dramatic compared to that of brain injury, and DR up-regulated NT-3 in the hippocampus. These findings suggest that DR is a novel stimulus paradigm that induces a pattern of NTF responses distinct from that of brain injury, and we therefore hypothesize that this may be a mechanism that increases the resistance of the brain to age-related neurodegenerative insults.

***Neurogenesis in adult brain.*** Unlike skin and liver, in which dead cells can be replaced by nearby mitotic cells, the brain apparently lacks this regenerative capacity, making it vulnerable to injury and disease. However, the nervous system contains populations of proliferative

**Table 1.** The altered expression of mRNAs encoding NTFs in the indicated regions of the brains from rats fed on a DR regimen compared to those of an AL regimen

| | Cortex | | Hippocampus | |
|---|---|---|---|---|
| | DR/AL (%) | Significance* | DR/AL (%) | Significance* |
| NGF | 125 | S | 120 | NS |
| BDNF | 133 | S | 117 | NS |
| GDNF | ND | ND | ND | ND |
| CNTF | 118 | NS | 128 | S |
| NT-3 | 95 | NS | 136 | S |
| NT-4 | ND | ND | ND | ND |

*$p < 0.05$
S: significant; NS: not significant; ND: not detectable
NGF: nerve growth factor; BDNF: brain-derived neurotrophic factor; GDNF: glial-derived neurotrophic factor; CNTF: Ciliary neurotrophic factor; NT-3: neurotrophin-3; NT-4: neurotrophin-4

cells called neural stem cells that are capable of dividing, migrating and differentiating into neurons and glia. In the adult brain, this process of neurogenesis occurs in the subventricular zone (SVZ) adjacent to the lateral ventricles, and in the subgranular zone of the dentate gyrus (Christie and Cameron, 2006). In these areas, there appears to be a continuous turnover of interneurons and granule cells, implying that newborn neurons replace dying cells. Therefore, the ability of neural stem cells to integrate into various brain regions offers hope for the development of restorative therapies for ischemic, traumatic, and degenerative brain diseases. There is some evidence that damage to granule cells can trigger the increased proliferation and recruitment of new granule cells from resident progenitors (Gould and Tanapat, 1997), and insults that cause neuronal loss in hippocampus, are accompanied by increased neurogenesis in the dentate subgranular zone (Bengzon *et al.*, 1997). Neuronal stem cells may provide regions such as the hippocampus with their ability to support local neurogenesis throughout life. However, there is no direct evidence that the degenerated neurons are functionally replaced by the new ones. Interestingly, very recent findings suggest that endogenous neural precursors can be induced in situ to differentiate into mature neurons, in regions of adult mammalian neocortex that do not normally undergo neurogenesis (Magavi *et al.*, 2000). Clearly, considerable further work is required to establish the molecular and cellular mechanisms controlling neurogenesis. A better understanding of neural stem cells may provide novel approaches for preventing and treating neurodegenerative disorders.

***DR and adult hippocampal neurogenesis.*** Successful therapy of traumatic and degenerative disorders that affect the adult central nervous system (CNS) in human will require the ability both to reduce the rate and extent of tissue injury, and to restore or replace destroyed tissue. Most current therapeutic strategies focus on the former because of less regenerative capacity of adult brain. However, the recent development of methods for cell imaging, labeling, and counting has made an accurate and quantitative assessment of *in vivo* adult neurogenesis possible. In addition, studies from several laboratories have begun to reveal some of factors that can influence the rate of proliferation, the extent of survival, the substrates of migration, and events controlling fate choice decisions (Gage *et al.*, 1998). In considering therapies for human adult neurological diseases based on cellular replacement, transplantation of terminally differentiated neural cells has been most widely tested. However, an alternative clinical approach is to activate the endogenous stem cells to proliferate, differentiate, and become functional. Neurogenesis in the dentate gyrus of the hippocampus is now well known to be responsive to brain injury, and to both mental and physical activity (Duman *et al.*, 2001; Nilsson *et al.*, 1999; van Praag *et al.*, 1999). Results from our laboratory and others have reported that DR can increase hippocampal neurogenesis in the brains of adult rats and mice (Fontan-Lozano *et al.*, 2008; Kitamura *et al.*, 2006; Lee *et al.*, 2000, 2002b). Moreover, we first suggested direct evidence showing that elevated levels of BDNF in DR regimen play an important role in the enhancement of hippocampal neurogenesis resulting from DR in heterozygous BDNF knockout (BDNF +/-) mice (Lee *et al.*, 2002a). The results from this study suggest that BDNF plays an important role in the regulation of the basal level of neurogenesis in dentate gyrus of adult mice and that BDNF contributes to the DR-enhanced hippocampal neurogenesis by promoting the survival of newly generated neurons. Interestingly, it has been suggested that DR-mediated enhancement of hippocampal neurogenesis might be, in contrast to that of other stimulation such as exercise, independent on NMDA receptor functions in the study with mice lacking NMDA receptor 1 subunit (Kitamura *et al.*, 2006). This finding suggests that the NMDA receptor's roles are important for the activation of cell proliferation which was shown in the exercise-mediated neurogenesis, but nor for the enhancement of cell survival in dentate gyrus by DR.

Taken together, the results from recent DR studies clearly suggest that DR is potentially an efficacious regimen not only for the prevention of neurodengerative disorders but also in therapeutic approaches through its regulation of endogenous neural stem cells in adult brain.

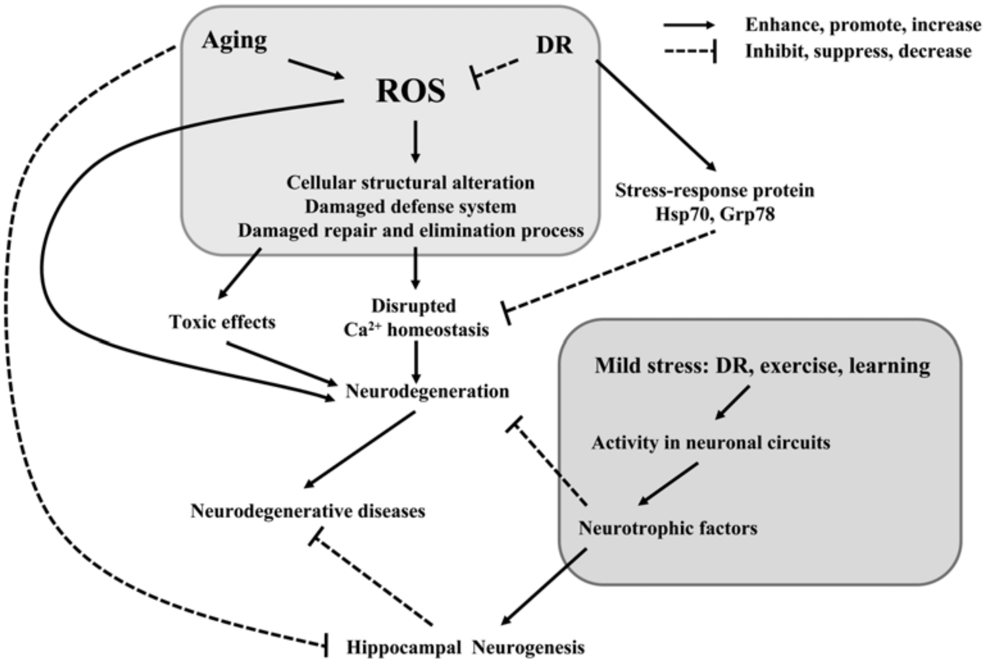

**Fig. 2.** A working model of the mechanisms whereby mild energy restriction protects the brain against neurodegenerative disorders, and stimulates neurogenesis in adult brain. It has been widely accepted that DR's anti-aging mechanisms are based on the free radical theory of aging (upper shaded box). Briefly, reactive oxygen species (ROS) are increased with age, and by multiple actions, could act to damage neurons. DR may suppress free radical generation. In the current study, we proposed that DR might also boost the expression of the cellular defense molecules such as stress response proteins (SRP) and neurotrophic factors (NTFs). Increased NTF signaling by DR as a mild stress, might change the capacity of endogenous neural stem cells, and provide a neuroprotective mechanism of DR (bottom opened box).

## CONCLUSION

Increasing numbers of studies in the effects of DR in aging and neurodegenerative diseases showed that neuronal survival and behavioral outcome are improved by caloric restriction in animal models of Alzheimer's, Parkinson's and Huntington's diseases, and stroke. However, the molecular and cellular mechanisms for the neuroprotective actions of DR have not been well established. Several studies have suggested that SRPs and NTFs are involved in the neuroprotective and neurorestorative effects of DR. Furthermore, the impact of DR on the granular progenitor cells in the adult hippocampus were also lately evaluated, and it was found that DR increased the levels of certain neurotrophic factors including BDNF and NT-3 in several different tissues of brain. When adult rats and mice are maintained on a DR feeding regimen, there is an increased expression of neurotrophins, as well as a greater number of newly generated cells in the dentate gyrus of the hippocampus, apparently as a result of increased cell survival. The majority of newborn cells in the granular layer of the hippocampus exhibit mature neuronal phenotypes, indicating enhanced hippocampal neurogenesis during a DR regimen. These findings clearly suggest that DR enhances hippocampal neurogenesis in adult brain through the increased NTFs signaling. Collectively, the current findings contribute to elucidating some of the molecular mechanisms underlying the neuroprotective actions of DR (Fig. 2). Furthermore, the current studies provide the basis for a possible preventative strategy whereby endogenous neural stem cells are recruited by dietary and/or pharmaceutical modulation to proliferate, migrate, and differentiate into appropriate functional neurons to deal with neuronal loss and damage in age-related neurodegenerative diseases.

## ACKNOWLEDGEMENTS

This work was supported for two years by Pusan National University Research Grant, 2007-2008.
